# An Unusual Association: Iliopsoas Bursitis Related to Calcium Pyrophosphate Crystal Arthritis

**DOI:** 10.1155/2015/935835

**Published:** 2015-10-15

**Authors:** Marco Di Carlo, Antonella Draghessi, Marina Carotti, Fausto Salaffi

**Affiliations:** ^1^Rheumatology Department, Polytechnic University of the Marche, Jesi, 60035 Ancona, Italy; ^2^Radiology Department, Polytechnic University of the Marche, 60035 Ancona, Italy

## Abstract

A 71-year-old man with osteoarthritis and chondrocalcinosis came to our observation developing a swelling in the groin region after a recent left colectomy for adenocarcinoma. The imaging techniques revealed the presence of an iliopsoas bursitis in connection with the hip. The synovial fluid analysis detected the presence of calcium pyrophosphate (CPP) crystals and allowed the final and unusual diagnosis of iliopsoas bursitis related to acute CPP crystal hip arthritis.

## 1. Introduction

Hip pain sometimes could represent a challenge even for expert clinicians and could require many imaging techniques efforts to complete the differential diagnostic workup. The hip is one of the most complex joints of the human body, surrounded by a significant number of ligaments and bursae that complicate the detection of the origin of a clinical problem. The iliopsoas bursa (also called iliopectineal) is one of the largest articular recesses of the human body [1]. It lies between the iliopsoas and pectineus muscles anteriorly and the iliopectineal eminence and hip capsule posteriorly. While in the normal subject iliopsoas bursa is a virtual cavity, in pathological conditions it could become a palpable mass. Usually a bursal enlargement is due to a hip joint illness and communications between joint and bursa have been described in the 15% of healthy subjects. The more frequent hip diseases that could determine an iliopsoas bursitis are represented by rheumatoid arthritis, osteoarthritis, osteonecrosis, synovial chondromatosis, pigmented villonodular synovitis, septic arthritis, and complications of total hip arthroplasty [2–11]. Potentially, any condition able to generate a joint effusion and determining an elevation of intra-articular pressure may involve the iliopsoas bursa, both in acute and in chronic damage. Between the proinflammatory stimuli in order to cause a hip arthritis are included CPP crystals [12]. The inflammation of the bursa results in a disabling pain in the groin region, with the hip kept in flexion and external rotation. Pain is enhanced by walking or by any action determining a joint extension. Bursa could be also painless and revealed on clinical examination as a soft tissue mass. Other manifestations of iliopsoas bursitis could be secondary to the ab extrinseco compression of the femoral and iliac vessels (with swelling of the thight or deep vein thrombosis of the leg), of the femoral nerve or of the bladder [13–16].

## 2. Case Report

**Figure 1:**
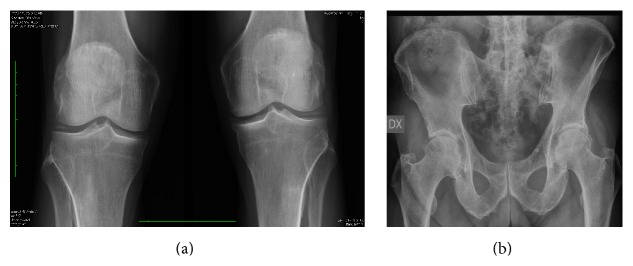
Conventional X-ray of knees (a) and hips (b). The figure shows a picture of osteoarthritis associated with calcifications in menisci (a) and in the soft tissues near the hip bilaterally (b).

A 71-year-old Caucasian man, with a history of osteoarthritis and knee chondrocalcinosis (Figure 1), never complicated with acute arthritis, arrived to our department for a recent onset of pain at right hip accompanied by homolateral swelling in the groin region. Two months earlier, he underwent surgery (left colectomy) for colic adenocarcinoma. The pain arose rapidly within two weeks and was determining a severe functional impotence of lower right limb. The clinical picture was completed by the presence of a low-grade fever (not higher than 37.2°C).

The physical examination revealed the swelling area that could easily be appreciated around and directly above the hip. The hip range of the movement was extremely limited. The patient was able to walk without crutches, keeping his right hip flexed and avoiding putting weight on it. The patellar and achilles reflexes were normal while the strength of quadriceps was decreased.

The laboratoristic parameters showed an augmented C-reactive protein (3.9 mg/dL, normal value <0.8), while erythrocyte sedimentation rate, anticitrulline antibodies, and rheumatoid factor level were normal as the screening for reactive arthritis.

**Figure 2:**
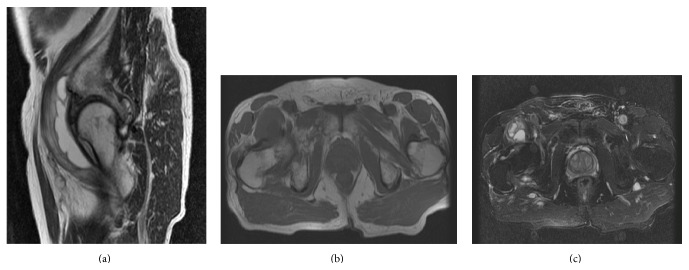
Magnetic resonance imaging (MRI). An elongated neoformation with predominantly fluid content is shown, multiloculated and localized in the anterior-lower aspect of the right hip joint. (a) Sagittal T2-weighted scan. (b) Axial T1-weighted magnetic resonance (MR) image. (c) Axial short tau inversion recovery (STIR) MR image.

**Figure 3:**
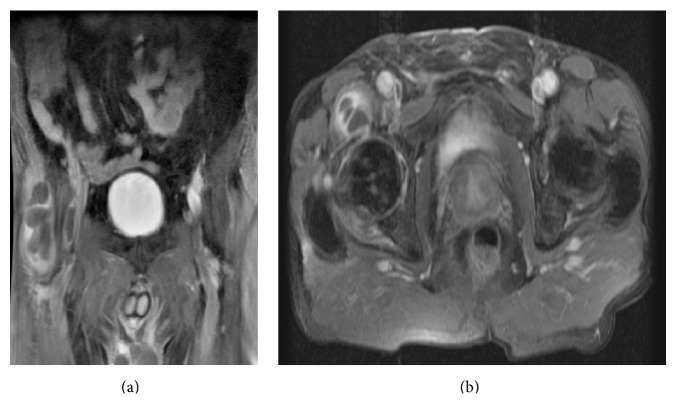
MRI with contrast. The MRI sequences with contrast medium confirm the predominantly fluid content of the neoformation, with contrast enhancement of the peripheral portion and the septa. Synovitis of the right hip is detectable. (a) Coronal postcontrast T1-weighted MR image with fat saturation. (b) Axial postcontrast T1-weighted MR image with fat saturation.

Magnetic resonance imaging (MRI) of the pelvis revealed an elongated neoformation with predominantly liquid content, multiloculated and localized in the anterior-lower aspect of the right hip joint, extending longitudinally for 6.5 cm and in diameter axial for 3.1 cm with multiple partitions in the context (Figure 2). The MRI sequences with contrast in coronal and axial scanning corroborated that the content is predominantly liquid, with enhancement contrast of the peripheral portion and the septa. These reliefs are associated with signs of synovitis of right hip (Figure 3).

**Figure 4:**
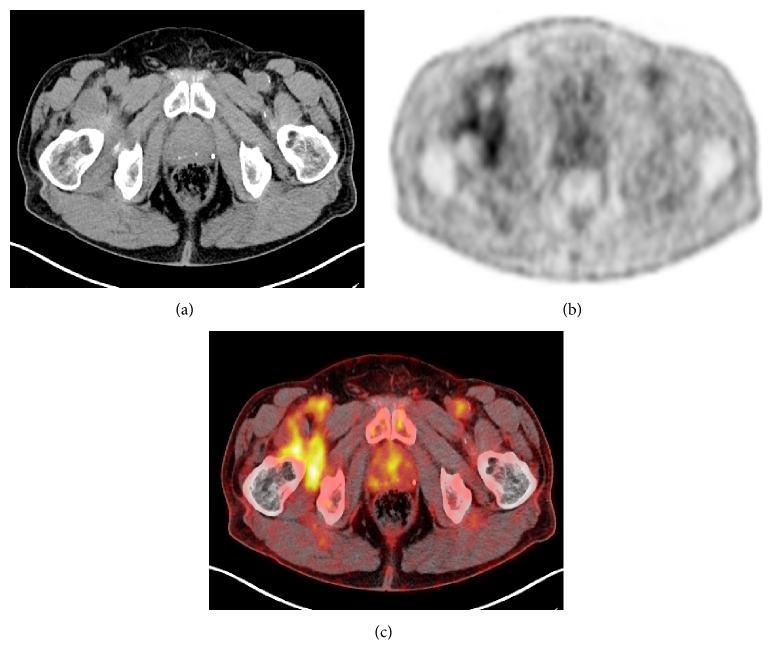
(a) Computerized tomography (CT) axial scan. (b) Fluorine-18-fluorodeoxyglucose positron emission tomography (^18^F-FDG PET) axial scan. (c) ^18^F-FDG-PET/CT fusion image. A moderate uptake in the anterior-lower aspect of the right hip joint is appreciable, more evident in the peripheral portion of the neoformation. A remarkable uptake is present in right hip joint.

Then, considering the persistent conundrum in differential diagnosis, with a strong suspicion of a metastatic origin, as requested by the colleagues, surgeons, the patient underwent a computerized tomography (CT) and a fluorine-18-fluorodeoxyglucose positron emission tomography (^18^F-FDG PET). In the fusion images (PET/CT), a moderate uptake of the radiopharmaceutical drug (18-fluorodeoxyglucose) in the anterior and inferior part of coxofemoral right is appreciated, more evident in the peripheral portion, and intense storage of the radioisotope in the hip joint intra-articular level (Figure 4).

**Figure 5:**
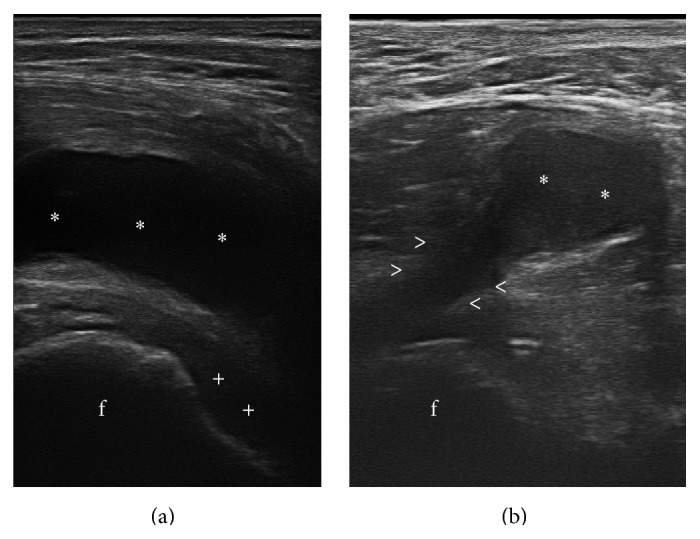
Ultrasound examination of the hip. (a) Longitudinal scan revealing the presence of a neoformation with fluid content (asterisks) anterior to the right hip. In the hip joint, an effusion (+) is present. (b) Semicoronal scan showing the connection between the joint and the formation (arrowheads), f: femoral head.

Finally, in order to obtain a sample of the bursal fluid collection, an ultrasound examination with a following guided aspiration was performed. Gray-scale and color Doppler sonography was performed with the latest generation machine (My Lab Twice HD, Esaote S.p.A., Genova, Italia), equipped with linear probe with broad frequency band (6–18 MHz) and the Doppler frequency of more than 9 MHz. Axial and sagittal images were obtained while the patient was in a supine position, putting the probe over the hip. In particular, the longitudinal scan demonstrated the presence of a predominantly liquid content before the right coxofemoral joint, associated with synovitis of the hip. Semicoronal scanning, instead, showed the presence of the connection between the hip joint and the neoformation (Figure 5).

A turbid fluid with diminished viscosity and inflammatory features (leukocytes count: 8600/mm^3^, normal value <200; proteins: 5.16 g/dL, normal value 1–2.5) was aspirated from the bursa. Surprisingly, the optical microscopy fluid analysis revealed the abundant presence of small rhomboid crystals with a weak positive birefringence.

The following cytological examination was negative for neoplastic cells and the bacteriological examinations did not detect any pathogen agent.

The final diagnosis was iliopsoas bursitis related to acute CPP crystal hip arthritis.

Colchicine in association with an oral steroid course determined a prompt remission of the articular and bursal inflammatory signs.

## 3. Discussion

Iliopsoas bursitis is usually due to an excessive production of synovial fluid in the hip. While the pathogenesis is not well known yet, it is postulated that the collection of fluid in the bursa is associated with a high pressure effusion inside the joint. Then, the fluid is pushed from the joint to the bursa, a zone with a lower pressure [17]. In this point of view, bursa represents a volume reservoir that protects the hip from an overload.

For other authors, the bursitis is a simple protrusion of the joint capsule weakened by degenerative changes [18]. Indeed, in the majority of cases, a chronic damage in the coxofemoral joint with a low degree of inflammation is present. An ultrasonographic study reported a prevalence of 2.2% of iliopsoas bursitis in patients with hip osteoarthritis [7].

Rheumatoid arthritis frequently underlies an enlargement of iliopsoas bursa [2–5]. In this pathology, the synovial proliferation inside the bursa could be the primary lesion.

In our case, we believe that the synovial fluid originated from the joint, considering the detection of CCP crystals inside the bursal fluid and the calcifications of the cartilage of the acetabular head. The inflammatory action of CCP crystals induced an acute production of synovial fluid and, then, the increased pressure inside the hip allowed the bursal collection. The inflammation induced by CCP crystals could be so powerful to produce a complete hip destruction within few weeks [19]. Even if acute CPP crystal arthritis is one of the most common causes of acute monoarthritis in the elderly, to our knowledge, it is the first report of iliopsoas bursitis related to acute CCP crystal hip arthritis. Al-Khodairy et al. in 1997 [20] reported a case of iliopsoas bursitis in a subject with pseudogout of the knee but they failed to aspirate liquid from the bursa.

In regard of the recent colic neoplasm, an intensive and complete diagnostic route was performed, starting from the imaging techniques. The other possibilities of this kind of swelling in the groin region considered were a metastasis, a lymphocele, a partially colliquative lymph node, or a hematoma.

Finally, the synovial fluid analysis avoided a new surgical intervention to resect the cyst, and the final diagnosis was established accordingly to the European League against Rheumatism recommendations for calcium pyrophosphate deposition [21].

In the evaluation of a patient with pain and/or swelling in the hip region and with symptoms related to the lower limb difficult to explain, the presence of an iliopsoas bursitis should be kept in mind.
